# Successful Removal of a Penile Constriction Ring in a 14-Year-Old Male

**DOI:** 10.1155/2009/916507

**Published:** 2009-11-02

**Authors:** Timothy K. Suttle, Blake Palmer, Jonathan E. Heinlen, Christopher C. Roth, William G. Reiner, Dominic Frimberger

**Affiliations:** Department of Urology, University of Oklahoma, 920 Stanton L Young Boulevard WP 3150, Oklahoma City, OK 73104, USA

## Abstract

Penile strangulation is a rarely described medical emergency, especially in the adolescent population. This case demonstrates the successful removal of a constricting metal ring from the penis of a 14-year-old male with a diamond blade equipped orthopedic oscillating saw while under ketamine anesthesia in the emergency department.

## 1. Case Report

A 14-year-old male presented to the emergency department with a finger sized “mood” ring on his penis down to the penoscrotal junction. He placed the ring three days prior which resulted in significant penile edema, thus preventing its removal. The patient attempted to remove the ring with pliers and later with an engraving tool, which resulted in minor lesions on the dorsal shaft skin.

**Figure 1:**
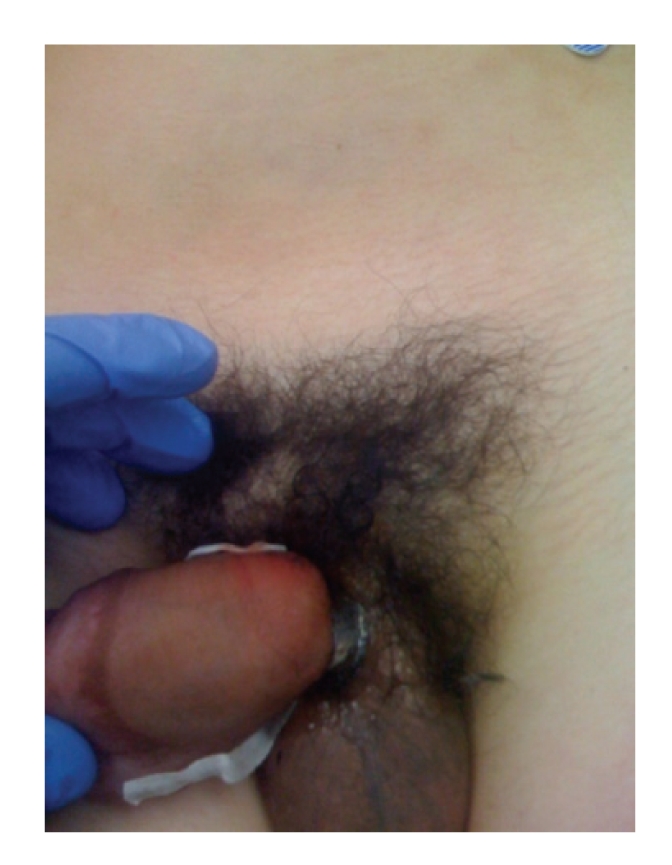
Patient at presentation, note distal penile edema.

**Figure 2:**
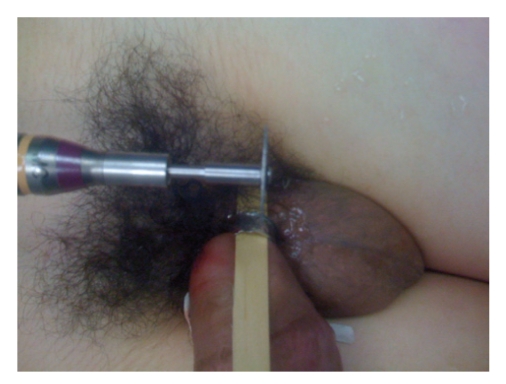
Illustration of tongue depressor placement and oscillating saw used for removal.

**Figure 3:**
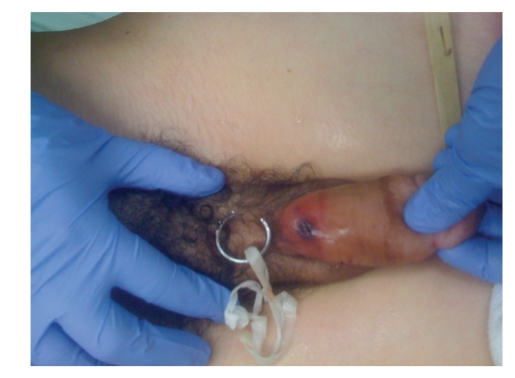
Patient following successful removal of ring.

Upon arrival he was in good condition, afebrile, and denied dysuria. On exam a finger-sized metal ring was noted at the base of the patient's penis with significant distal edema (Figure 1). After an initial attempt at removal with a ring cutter was unsuccessful due to the ring's width, an orthopedic oscillating saw equipped with a diamond blade was utilized for removal. In the emergency department the patient was placed under ketamine anesthesia and monitored by the ED attending. Under anesthesia, a wooden tongue depressor was placed between the ring and the patient's penis to protect the skin (Figure 2). The diamond blade saw was then used to cut the ring after which it was possible to bend, and remove it from the penis (Figure 3). Following removal, the distal edema began to resolve immediately and no clear necrosis or iatrogenic damage to the phallus was noted.

Child psychiatry was consulted, it was determined that the patient placed the ring on his penis for autoerotic purposes and was not deemed to be a threat to himself. The patient was also advised that it would be unnecessary to report this event in any future medical history taking. Due to the uniquely stressful nature of the incident, both the patient and his family were counseled prior to release to ensure the wellbeing of all parties. He was discharged the following day, after voiding with no postvoid residual.

## 2. Discussion

The motivation for intentional placement of penile constriction devices is variable depending on the patient's age and is not well described in the adolescent population. The adult population frequently reports erotic or autoerotic goals when intentionally placing constricting devices [1] as was the case with our patient. Pediatric patients may present with either accidental or intentional placement of a strangulating object, most commonly strands of hair [2]. The most often reported cause of children, or their guardians, intentionally placing hair around the penis is to prevent enuresis. Regardless of the means or rationale by which one decides to place a constricting device around the penis, strangulation is a urologic emergency and expeditious decompression is vital for a favorable outcome.

Numerous methods have been described for removal of constricting devices. When choosing a method one must take into account the material to be removed, severity of penile injury, and availability of tools. With metallic bands that cannot be removed with conventional ring cutters the string technique in concert with penile aspiration may be utilized [3]. Power-driven cutting tools include dremmel saws [4] and oscillating orthopedic saws [5] have been employed with excellent results, often avoiding the need for surgical intervention. However, if the penis is gangrenous, necrotic, or other modalities have failed, degloving, or amputation of the penis may be indicated contingent on the extent of devitalized tissue.

## 3. Conclusion

This case highlights the successful use of an orthopedic oscillating saw to remove a metal penile constriction ring in a 14-year-old male three days after placement.

##  Conflict of Interest Statement

The authors have no potential conflicts of interest. There were no outside sources of support or funding. There were no violations of the ethical policy of the journal.
